# Association between the prognostic nutritional index and cognitive impairment in patients with heart failure: a cross-sectional study

**DOI:** 10.3389/fnut.2026.1939333

**Published:** 2026-10-26

**Authors:** Chengyang Li, Yingxuan Gao, Qi Niu, Liangwan Chen, Yanjuan Lin

**Affiliations:** 1 School of Nursing, Fujian Medical University, Fuzhou, Fujian, China; 2 Department of Nursing, Fujian Medical University Union Hospital, Fuzhou, Fujian, China; 3 Department of Cardiac Surgery, Fujian Medical University Union Hospital, Fuzhou, Fujian, China; 4 Fujian Provincial Special Reserve Talents Laboratory, Fuzhou, Fujian, China

**Keywords:** cognitive impairment, cross-sectional association, heart failure, Montreal Cognitive Assessment, prognostic nutritional index

## Abstract

**Background:**

The prognostic nutritional index (PNI), calculated from serum albumin and peripheral lymphocyte count, is commonly interpreted as a nutritional–inflammatory marker. This study investigated the association between the PNI and cognitive impairment (CI) among patients with heart failure (HF).

**Methods:**

This cross-sectional study included 153 patients with HF. Before cognitive status classification and statistical analysis, one point was added to the original Montreal Cognitive Assessment (MoCA) total score for participants with fewer than 12 years of education. Participants were classified into the CI group (education-corrected MoCA <26) or the non-CI group (education-corrected MoCA ≥26).

**Results:**

CI was identified in 93 patients (60.8%). The median PNI was lower in the CI group than in the non-CI group [44.10 (40.55–47.00) vs. 47.08 (44.62–50.28), *p* < 0.001]. After adjusting for age, sex, and educational attainment, each five-point increase in the PNI was associated with lower odds of prevalent CI (odds ratio (OR): 0.562, 95% confidence interval (95% CI): 0.348–0.910; *p* = 0.019). The base clinical model had an area under the curve (AUC) of 0.868, and the PNI-extended model had an AUC of 0.882; the increment was small and non-significant (ΔAUC: 0.014, 95% CI: −0.008 to 0.036; DeLong *p* = 0.204). In a sensitivity analysis, albumin was independently associated with CI (OR: 0.362 per 5-g/L increase, 95% CI: 0.172–0.759; *p* = 0.007), and the PNI-extended model did not outperform the albumin-extended model (AUC: 0.882 vs. 0.887; ΔAUC: −0.005, 95% CI: −0.022 to 0.013; DeLong *p* = 0.588).

**Conclusion:**

Lower PNI was independently associated with higher odds of prevalent CI in patients with HF. However, its incremental discrimination beyond age, sex, and educational attainment was small and statistically non-significant, and the PNI-extended model did not outperform the albumin-extended model in the sensitivity analysis. These findings support an association but do not establish incremental clinical utility; the decision-curve analysis is exploratory, and external validation is required.

## 1 Introduction

Cognitive impairment is an important comorbidity in patients with HF. A recent global meta-analysis estimated that approximately 43% of older adults with HF have CI (1), and affected patients may experience poorer self-care, medication adherence, quality of life, and clinical outcomes (2–4). The MoCA is sensitive to mild cognitive deficits and is widely used to identify CI in patients with HF (2, 5). Nonetheless, routine cognitive testing is not universally available, creating interest in inexpensive biomarkers that may help identify patients who warrant formal cognitive assessments.

Malnutrition and systemic inflammation frequently coexist in patients with HF and may contribute to cognitive dysfunction through reduced physiologic reserve, endothelial dysfunction, immune activation, and impaired cerebral perfusion (2, 6–8). The prognostic nutritional index (PNI) is calculated from serum albumin and peripheral lymphocyte count and is often used as a nutritional–inflammatory marker. It is not, however, a direct or nutrition-specific measure: albumin is influenced by inflammation, hepatic synthesis, congestion, fluid status, and disease severity, whereas lymphocyte count is influenced by immune activation, infection, medications, and physiologic stress. Lower PNI and related nutritional deficits have been associated with mortality and rehospitalization in acute and chronic HF cohorts (9–18). Their associations with CI in patients with HF have received less attention.

The primary objective of this study was to examine whether the PNI differed between HF patients with MoCA-defined CI and those without MoCA-defined CI and whether the PNI remained independently associated with prevalent CI after adjustment for clinically relevant covariates.

## 2 Materials and methods

### 2.1 Study design and participants

This cross-sectional observational study included adult patients with clinically diagnosed HF who received care at Fujian Medical University Union Hospital between February and May 2025. Potentially eligible patients were screened from the inpatient cardiology ward, with 189 individuals initially identified through an electronic medical record-based screening list. Participant recruitment and exclusions are summarized in Figure 1. Of the 189 patients initially identified, 21 declined participation, and 168 underwent an eligibility assessment. After excluding 15 patients, 153 patients were enrolled and included in the final analysis. Eligible participants were required to (1) be aged ≥18 years and (2) have a diagnosis of HF according to the 2024 Chinese Guidelines for the Diagnosis and Treatment of Heart Failure. The exclusion criteria were as follows: (1) patients with concomitant gastrointestinal diseases, including gastrointestinal malignancies, Crohn’s disease, inflammatory bowel disease, or ulcerative colitis; (2) patients with neurological disorders, such as Alzheimer’s disease or Parkinson’s disease; (3) patients with psychiatric disorders, such as depression or schizophrenia; (4) patients with a history of cancer; and (5) individuals unable to cooperate with the cognitive assessment due to severe visual or auditory impairments or similar conditions.

**Figure 1:**
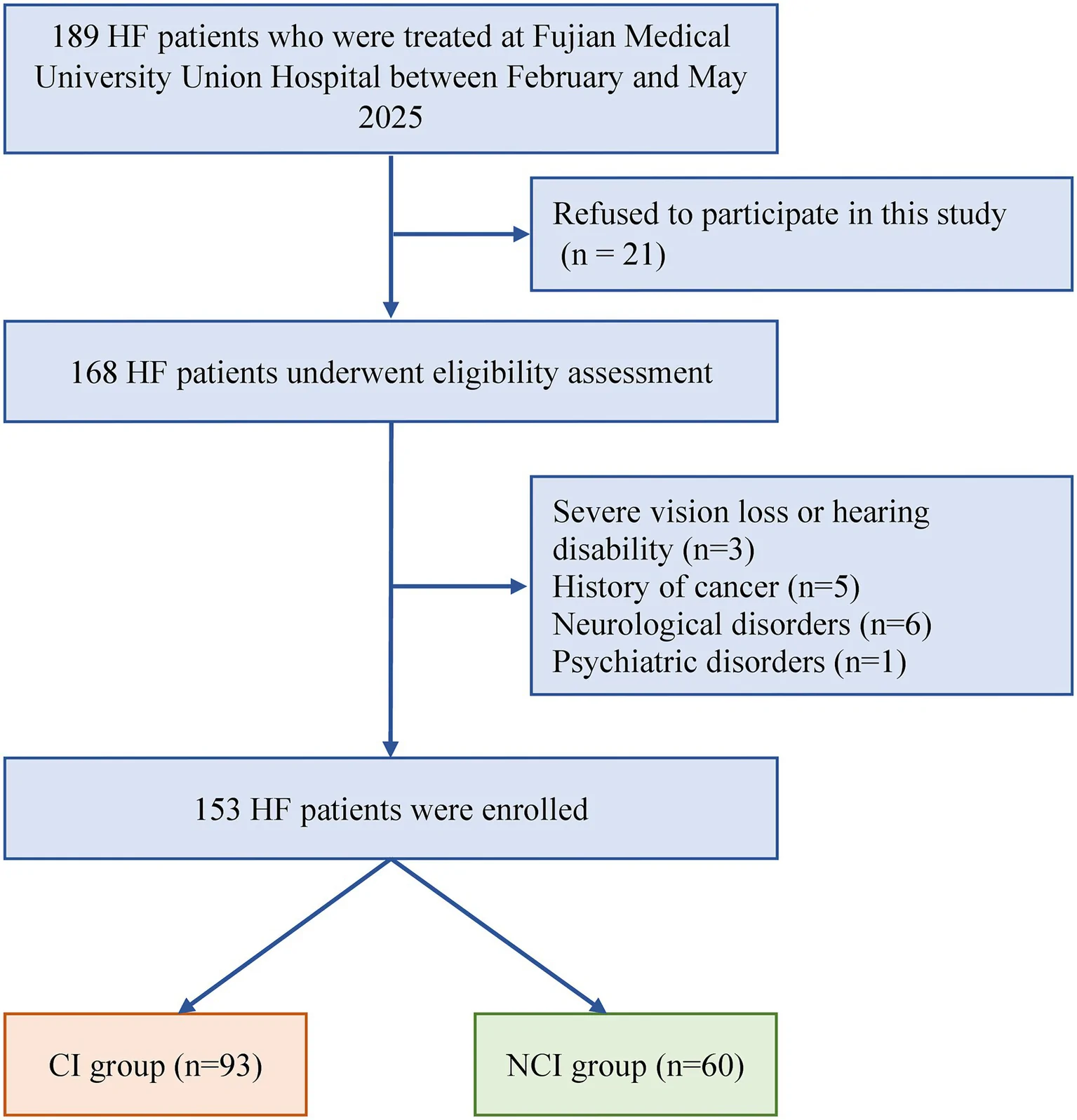
Participant flow and cognitive grouping. Of the 189 HF patients, 153 were included and classified using education-corrected MoCA scores into the CI group (MoCA <26; *n* = 93) and the NCI group (MoCA ≥ 26; *n* = 60). HF, heart failure; CI, cognitive impairment; NCI, non-cognitive impairment.

The study protocol was approved by the Ethics Committee of Fujian Medical University Union Hospital (approval no. 2025KY027). Written informed consent for participation was obtained from each participant before enrollment. The study was conducted in accordance with the Declaration of Helsinki.

### 2.2 Clinical and laboratory variables

The analysis dataset included age, sex, educational attainment level, height, weight, smoking history, diabetes mellitus, hypertension, coronary artery disease, New York Heart Association (NYHA) class, left ventricular ejection fraction (LVEF), NT-proBNP, cardiac troponin I, complete blood count indices, serum albumin, renal function indices, glucose, potassium, and sodium. All 153 participants were included in the reported analyses. Body mass index was calculated as weight in kilograms divided by height in meters squared. For the regression analyses, educational attainment was categorized as junior high school or below vs. senior high school or above. For exploratory analyses, the HF phenotype was classified according to the available LVEF measurements as heart failure with reduced ejection fraction (HFrEF; LVEF ≤40%), heart failure with mildly reduced ejection fraction (HFmrEF; LVEF 41–49%), or heart failure with preserved ejection fraction (HFpEF; LVEF ≥50%).

### 2.3 PNI calculation

The PNI was calculated as serum albumin (g/L) + 5 × absolute lymphocyte count (×10^9^/L), consistent with recent HF studies and evidence syntheses (9, 10). Observed PNI values match the formula to numerical precision. The PNI was interpreted as a composite nutritional–inflammatory marker rather than a direct measure of nutritional status because both serum albumin and lymphocyte count are affected by non-nutritional clinical processes. Because serum albumin and lymphocyte count are exact components of the PNI, they were not entered simultaneously with the PNI in the same multivariable model.

### 2.4 Cognitive assessment

Cognitive status was determined using the MoCA total score (2, 5). In accordance with the prespecified scoring procedure, one point was added to each participant’s original MoCA total score when their educational attainment was less than 12 years. This correction was applied before derivation of the binary cognitive outcomes and before all statistical analyses. CI was defined as an education-corrected MoCA score of <26, whereas non-CI was defined as a score of ≥26.

### 2.5 Statistical analysis

All statistical analyses were performed using the IBM Statistical Package for Social Sciences (SPSS, version 26.0; IBM, NY, USA) and R software (version 4.3.3). The normality of continuous variables was assessed using the Shapiro–Wilk test. Normally distributed variables are presented as mean ± standard deviation and were compared using a Welch’s independent-samples *t*-test; non-normally distributed variables are presented as the median (interquartile range) and were compared using the Mann–Whitney *U*-test. Categorical variables are expressed as numbers (percentages) and were compared using the chi-squared test or Fisher’s exact test, as appropriate.

A binary logistic regression analysis was performed to identify factors independently associated with CI, with results reported as ORs and 95% CIs. The primary multivariable logistic regression model was specified to evaluate the independent association between the PNI and prevalent CI. The model included PNI, age, sex, and educational attainment on the basis of clinical relevance rather than statistical significance in between-group or univariable analyses. Univariable models were used to describe crude associations, but they were not used to screen variables. Because serum albumin and lymphocyte count are algebraic components of the PNI, they were not entered separately into the PNI-focused multivariable model. As a sensitivity analysis, a clinically expanded PNI model additionally included body mass index, coronary artery disease, LVEF, and NYHA class III–IV. The potential effect modification by the HF phenotype was evaluated by including an interaction term between the PNI and the HF phenotype in the adjusted logistic regression model; this interaction analysis was exploratory because the study was not powered for phenotype-specific inference. The PNI was analyzed per five-point increase, age per a 10-year increase, LVEF per a 10-percentage-point increase, hemoglobin per a 10-g/L increase, and albumin per a 5-g/L increase. Multicollinearity among the retained predictors was assessed using variance inflation factors (VIFs).

A receiver operating characteristic (ROC) curve analysis was performed to evaluate model discrimination. To assess the incremental contribution of the PNI, a base clinical model comprising age, sex, and educational attainment was compared with a PNI-extended model. As an additional sensitivity analysis, we assessed whether the composite PNI provided information beyond albumin by comparing PNI-only and albumin-only models and by comparing the PNI-extended model with an alternative model comprising age, sex, educational attainment, and albumin. The PNI and albumin were evaluated in alternative rather than simultaneous models because albumin is an algebraic component of the PNI. AUCs and 95% CIs were estimated, and paired AUC differences were evaluated using the DeLong test. Nested models were additionally compared using likelihood-ratio tests, and Brier scores were reported as measures of overall prediction error. Decision-curve analysis was used only as an exploratory assessment of potential net benefit. The threshold probability was defined as the minimum estimated probability of prevalent CI at which referral for formal cognitive assessment would be considered warranted. Because no universally accepted referral threshold exists, interpretation was restricted to a clinically motivated probability range of 0.20–0.70; extreme thresholds were not interpreted because of the instability associated with the modest sample size. Internal validation was performed using 1,000 bootstrap resamples. The mean bootstrap optimism was subtracted from the apparent AUC to obtain an optimism-corrected estimate. Calibration was evaluated using the Brier score, mean absolute calibration error, the Hosmer–Lemeshow goodness-of-fit test, and a bootstrap-corrected calibration curve. Bootstrap correction represents internal validation and does not establish external transportability. All tests were two-sided, and a *p*-value of less than 0.05 was considered statistically significant.

## 3 Results

### 3.1 Baseline characteristics

Among the 153 patients, 93 (60.8%) had MoCA-defined CI and 60 (39.2%) did not have MoCA-defined CI. Patients with CI were older, more likely to be women, less likely to have a senior high school education or above, and had a lower body mass index. Coronary artery disease was more frequent in the cognitive impairment group. The median PNI was 44.10 (IQR 40.55–47.00) in the cognitive impairment group and 47.08 (44.62–50.28) in the non-CI group (*p* < 0.001; Table 1). Among patients with CI, 48 (51.6%) had HFrEF, 16 (17.2%) had HFmrEF, and 29 (31.2%) had HFpEF. The corresponding numbers in the non-CI group were 39 (65.0%), 9 (15.0%), and 12 (20.0%), respectively. The overall HF phenotype distribution did not differ significantly between the groups (*χ*^2^ = 2.96; *p* = 0.228).

**Table 1:** Comparison of clinical parameters between HF patients with CI and those without CI.

Variables	CI group (*n* = 93)	NCI group (*n* = 60)	Statistic	*p*-value
Age, years	68.03 ± 9.11	53.93 ± 10.82	*t* = 8.36	<0.001
Female, *n* (%)	39 (41.9%)	13 (21.7%)	*χ*^2^ = 6.68	0.010
Senior high school or above, *n* (%)	13 (14.0%)	17 (28.3%)	*χ*^2^ = 5.27	0.022
Body mass index, kg/m^2^	22.04 (20.20, 25.40)	24.83 (22.51, 27.09)	*U* = 1751.00	<0.001
Smoking history, *n* (%)	29 (31.2%)	29 (48.3%)	*χ*^2^ = 4.56	0.033
Diabetes mellitus, *n* (%)	27 (29.0%)	10 (16.7%)	*χ*^2^ = 3.04	0.081
Hypertension, *n* (%)	46 (49.5%)	21 (35.0%)	*χ*^2^ = 3.10	0.078
Coronary artery disease, *n* (%)	24 (25.8%)	6 (10.0%)	*χ*^2^ = 5.78	0.016
NYHA class III–IV, *n* (%)	69 (74.2%)	41 (68.3%)	*χ*^2^ = 0.62	0.431
LVEF, %	38.70 (28.20, 53.20)	29.55 (22.10, 44.17)	*U* = 3054.50	0.005
NT-proBNP	2447.00 (893.00, 6829.00)	2044.00 (575.00, 3841.50)	*U* = 3074.00	0.079
Cardiac troponin I	0.02 (0.01, 0.07)	0.02 (0.01, 0.06)	*U* = 1056.50	0.901
White blood cell count, ×10^9^/L	5.68 (4.78, 7.40)	6.48 (5.38, 7.76)	*U* = 2404.00	0.150
Neutrophils, %	64.70 (55.40, 71.00)	60.95 (54.68, 69.97)	*U* = 3125.50	0.211
Lymphocyte count, ×10^9^/L	1.49 (1.12, 1.83)	1.67 (1.22, 1.98)	*U* = 2232.00	0.047
Hemoglobin, g/L	127.00 (110.00, 136.00)	142.00 (131.00, 150.00)	*U* = 1569.50	<0.001
Platelet count, ×10^9^/L	179.00 (147.00, 222.00)	194.00 (159.50, 224.75)	*U* = 2457.50	0.215
Albumin, g/L	37.10 (34.20, 38.80)	38.70 (37.00, 40.82)	*U* = 1714.00	<0.001
Urea	7.10 (5.57, 10.03)	6.70 (5.35, 7.70)	*U* = 3104.00	0.195
Creatinine	85.00 (66.00, 118.00)	79.50 (73.50, 98.25)	*U* = 2933.00	0.594
Glucose	5.12 (4.32, 6.93)	4.66 (4.25, 5.83)	*U* = 3173.50	0.120
Potassium, mmol/L	3.92 (3.69, 4.31)	3.97 (3.73, 4.27)	*U* = 2599.00	0.586
Sodium, mmol/L	139.96 ± 3.38	139.75 ± 3.30	*t* = 0.38	0.705
Prognostic nutritional index	44.10 (40.55, 47.00)	47.08 (44.62, 50.28)	*U* = 1603.50	<0.001
HFrEF, *n* (%)	48 (51.6%)	39 (65.0%)	*χ*^2^ = 2.96	0.228
HFmrEF, *n* (%)	16 (17.2%)	9 (15.0%)		
HFpEF, *n* (%)	29 (31.2%)	12 (20.0%)		

Data are mean +/− SD, median (interquartile range), or *n* (%). *p*-values were based on the complete analysis dataset. CI, cognitive impairment; NCI, non-cognitive impairment; LVEF, left ventricular ejection fraction; NYHA, New York Heart Association; PNI, prognostic nutritional index.

### 3.2 Logistic regression analysis

Univariable logistic regression analyses were used to describe the crude associations between the candidate variables and prevalent CI. Each five-point increase in the PNI was associated with lower unadjusted odds of CI (OR, 0.410; 95% CI, 0.272–0.618; *p* < 0.001). These univariable results were not used to select covariates for the primary adjusted model (Table 2).

**Table 2:** Crude and adjusted associations with prevalent CI in patients with HF.

Variable	Univariable		Primary model		Sensitivity model	
	OR (95% CI)	*p*-value	OR (95% CI)	*p*-value	OR (95% CI)	*p*-value
Age, per 10 years	4.270 (2.627–6.942)	<0.001	4.332 (2.505–7.489)	<0.001	4.140 (2.328–7.362)	<0.001
Female vs. male	2.611 (1.246–5.470)	0.011	3.146 (1.180–8.386)	0.022	2.512 (0.918–6.875)	0.073
Senior high school or above	0.379 (0.169–0.848)	0.018	0.346 (0.125–0.957)	0.041	0.346 (0.119–1.009)	0.052
Body mass index, per 1 kg/m^2^	0.843 (0.763–0.931)	<0.001	–	–	0.886 (0.775–1.012)	0.075
Smoking history	0.484 (0.248–0.947)	0.034	–	–	–	–
Coronary artery disease	3.130 (1.195–8.199)	0.020	–	–	1.475 (0.428–5.083)	0.538
LVEF, per 10 percentage points	1.296 (1.043–1.609)	0.019	–	–	1.011 (0.756–1.350)	0.944
NYHA class III–IV	1.332 (0.652–2.724)	0.432	–	–	1.834 (0.642–5.239)	0.257
Lymphocyte count, per 1 × 10^9^/L	0.460 (0.262–0.808)	0.007	–	–	–	–
Hemoglobin, per 10 g/L	0.679 (0.561–0.822)	<0.001	–	–	–	–
Albumin, per 5 g/L	0.296 (0.165–0.531)	<0.001	–	–	–	–
PNI, per 5 points	0.410 (0.272–0.618)	<0.001	0.562 (0.348–0.910)	0.019	0.596 (0.364–0.977)	0.040

ORs and 95% CIs were estimated using logistic regression analyses. The primary model included age, sex, educational attainment, and the PNI. The sensitivity model additionally included body mass index, coronary artery disease, LVEF, and NYHA class. Serum albumin and lymphocyte count were not entered together with the PNI because they are algebraic components of the PNI. CI, confidence interval; LVEF, left-ventricular ejection fraction; NYHA, New York Heart Association; OR, odds ratio; PNI, prognostic nutritional index.

In the clinically informed primary model, which was adjusted for age, sex, and educational attainment, each five-point increase in the PNI was associated with lower odds of prevalent CI (OR: 0.562, 95% CI: 0.348–0.910; *p* = 0.019). This association remained evident in the expanded PNI sensitivity model, additionally adjusted for body mass index, coronary artery disease, LVEF, and NYHA classes III–IV (OR: 0.596, 95% CI: 0.364–0.977; *p* = 0.040). In a separate alternative model, which was adjusted for age, sex, and educational attainment, each 5-g/L increase in albumin was independently associated with lower odds of prevalent CI (OR: 0.362, 95% CI: 0.172–0.759; *p* = 0.007). Because albumin is a component of PNI, these alternative-model estimates should not be interpreted as mutually adjusted effects. No evidence of problematic multicollinearity was observed. There was no evidence that the association between the PNI and prevalent CI differed across HF phenotypes (*p* for interaction = 0.586); this interaction analysis was exploratory and limited by the small phenotype-specific subgroup sizes.

### 3.3 Internal model performance and decision-curve analysis

The base clinical model comprising age, sex, and educational attainment had an apparent AUC of 0.868 (95% CI: 0.810–0.927). After the addition of the PNI, the apparent AUC was 0.882 (95% CI: 0.827–0.938). The absolute AUC difference was 0.014 (95% CI: −0.008 to 0.036) and was not statistically significant (paired DeLong *p* = 0.204). The addition of the PNI improved overall model fit (likelihood-ratio *χ*^2^ = 6.20; *p* = 0.013) and reduced the Brier score from 0.141 to 0.132, but these findings do not establish incremental clinical utility. In the albumin sensitivity analysis, the PNI alone did not outperform albumin alone (AUC: 0.713 vs. 0.693; ΔAUC: 0.020, 95% CI: −0.037 to 0.077; DeLong *p* = 0.497). Similarly, the PNI-extended model did not outperform the albumin-extended model (AUC: 0.882 vs. 0.887; ΔAUC: −0.005, 95% CI: −0.022 to 0.013; DeLong *p* = 0.588; Table 3; Figure 2). These estimates represent internal model performance and should not be interpreted as externally validated predictive accuracy.

**Table 3:** Internal performance of the base, albumin-extended, and PNI-extended models for identifying prevalent CI in patients with HF.

Model	Apparent AUC (95% CI)	Optimism-corrected AUC	Brier score	ΔAUC (95% CI)	DeLong *p*
Base clinical model: age + sex + education	0.868 (0.810–0.927)	0.858	0.141	Reference	–
Albumin-extended model: age + sex + education + albumin	0.887 (0.834–0.941)	0.874	0.130	0.019 (−0.006 to 0.043)	0.133
PNI-extended model: age + sex + education + PNI	0.882 (0.827–0.938)	0.869	0.132	0.014 (−0.008 to 0.036)	0.204

The base model included age, sex, and educational attainment. The albumin-extended and PNI-extended models added albumin and the PNI, respectively, in alternative models. Optimism-corrected AUCs were estimated using 1,000 bootstrap resamples. Compared to the base model, the albumin-extended model had ΔAUC 0.019 (95% CI: −0.006 to 0.043; DeLong *p* = 0.133; likelihood-ratio *χ*^2^ = 8.36, *p* = 0.004), and the PNI-extended model had ΔAUC 0.014 (95% CI: −0.008 to 0.036; DeLong *p* = 0.204; likelihood-ratio *χ*^2^ = 6.20, *p* = 0.013). Directly comparing the PNI-extended model to the albumin-extended model yielded an ΔAUC of −0.005 (95% CI: −0.022 to 0.013; DeLong *p* = 0.588). AUC, area under the curve; ROC, receiver operating characteristic; CI, confidence interval; PNI, prognostic nutritional index.

**Figure 2:**
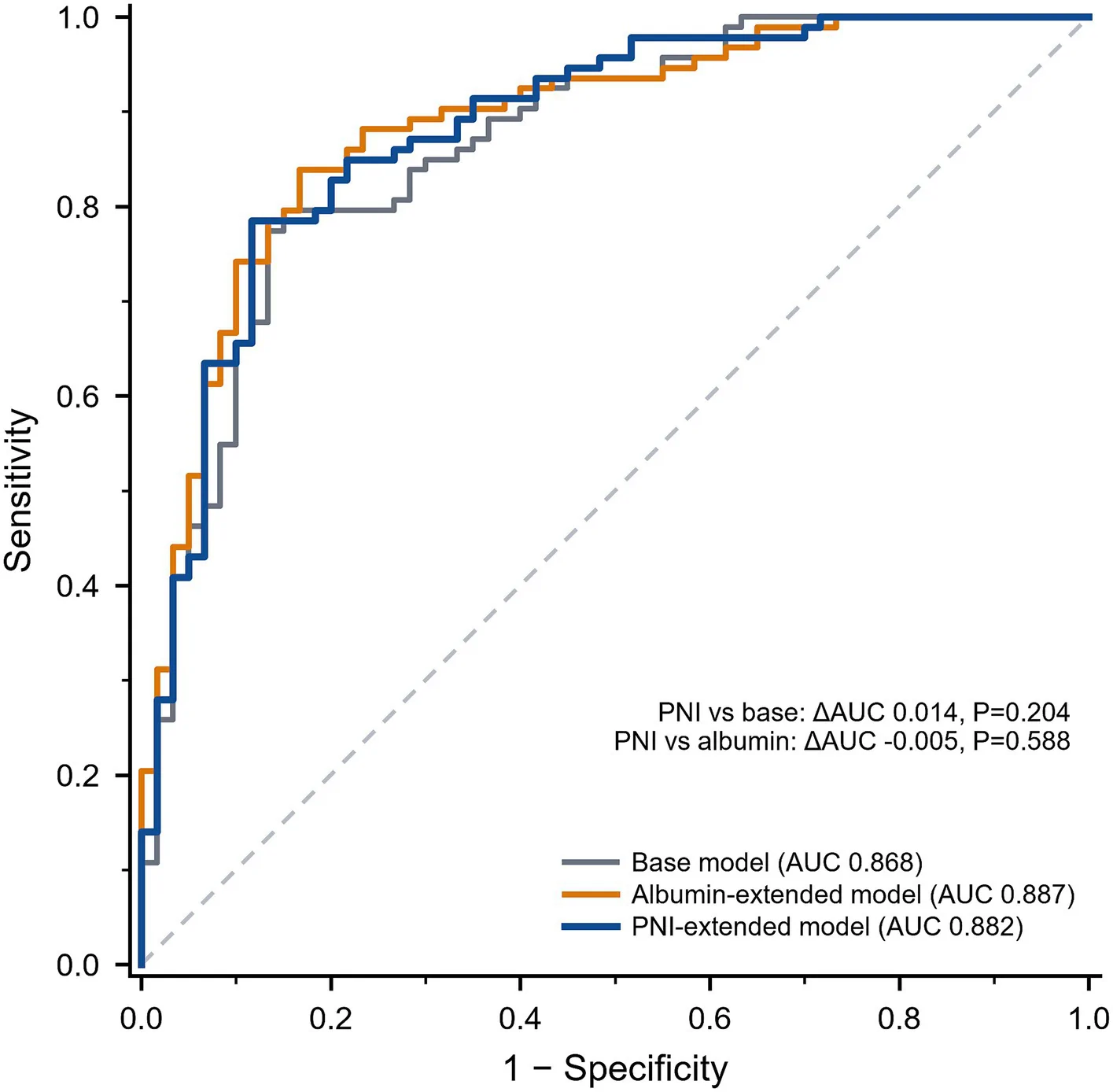
ROC curves of the base, albumin-extended, and PNI-extended models. The base model included age, sex, and educational attainment (AUC = 0.868). Albumin and the PNI were added in alternative models (AUC = 0.887 and AUC = 0.882, respectively). PNI vs. base: ΔAUC 0.014 (95% CI: −0.008 to 0.036; DeLong *p* = 0.204). PNI vs. albumin: ΔAUC −0.005 (95% CI: −0.022 to 0.013; DeLong *p* = 0.588).

Within threshold probabilities of 0.20–0.70, the PNI-extended model was estimated to provide greater net benefit than the treat-all and treat-none strategies over portions of the range. Its estimated advantage over the base clinical model was small and non-uniform. The decision-curve analysis was therefore exploratory and does not establish clinical utility (Figure 3). After bootstrap correction for optimism, the AUC was 0.858 for the base clinical model and 0.869 for the PNI-extended model. The PNI-extended model had a Brier score of 0.132 and a mean absolute calibration error of 0.061. The Hosmer–Lemeshow test showed no evidence of a lack of fit (*χ*^2^ = 7.80; *df* = 8; *p* = 0.454), and the bootstrap-corrected calibration curve remained close to the ideal line (Figure 4). These findings describe internal performance and should not be interpreted as external validation.

**Figure 3:**
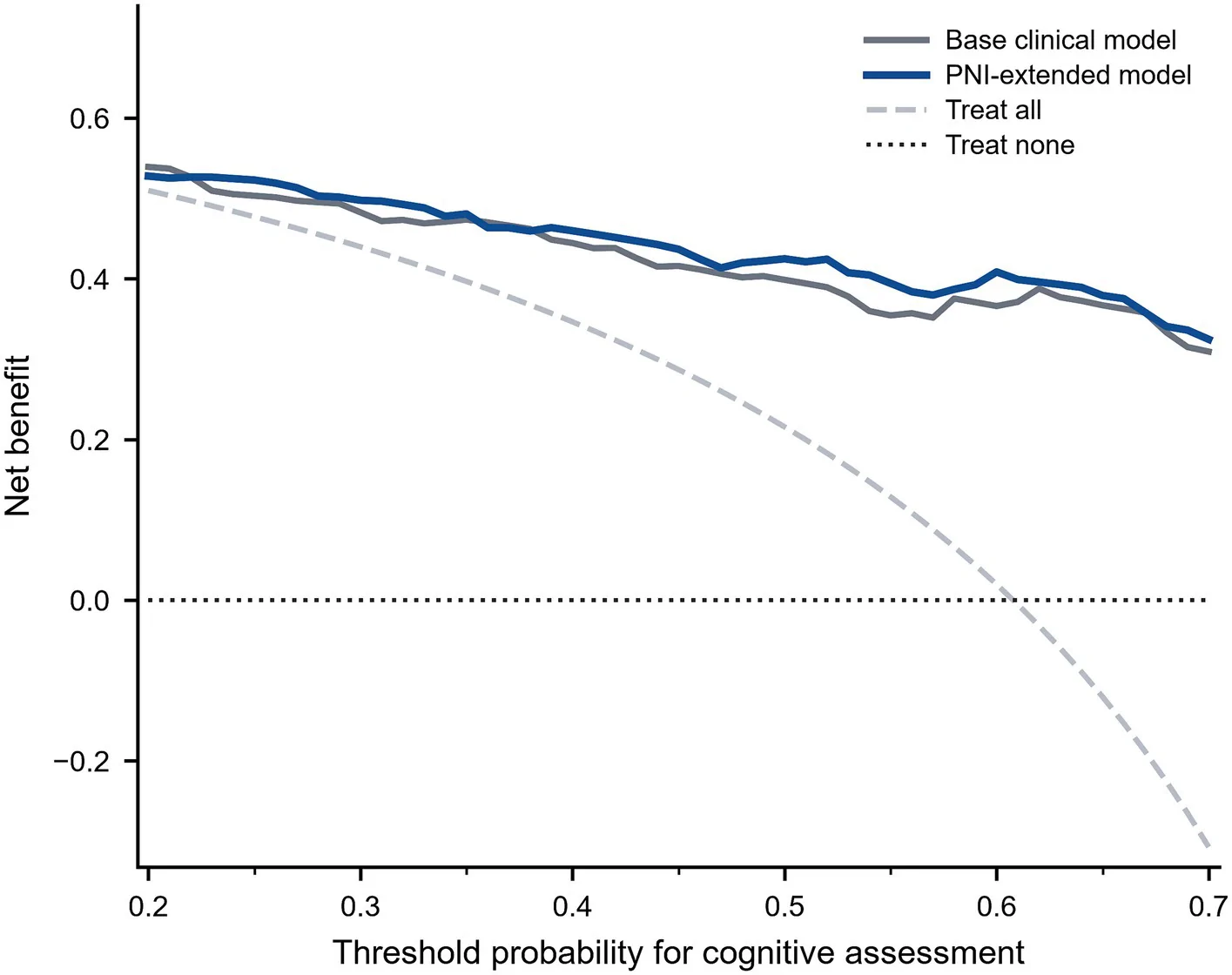
Exploratory decision-curve analysis of the base and PNI-extended models. Net benefit was compared across threshold probabilities of 0.20–0.70. The estimated advantage of the PNI-extended model over the base model was modest and non-uniform.

**Figure 4:**
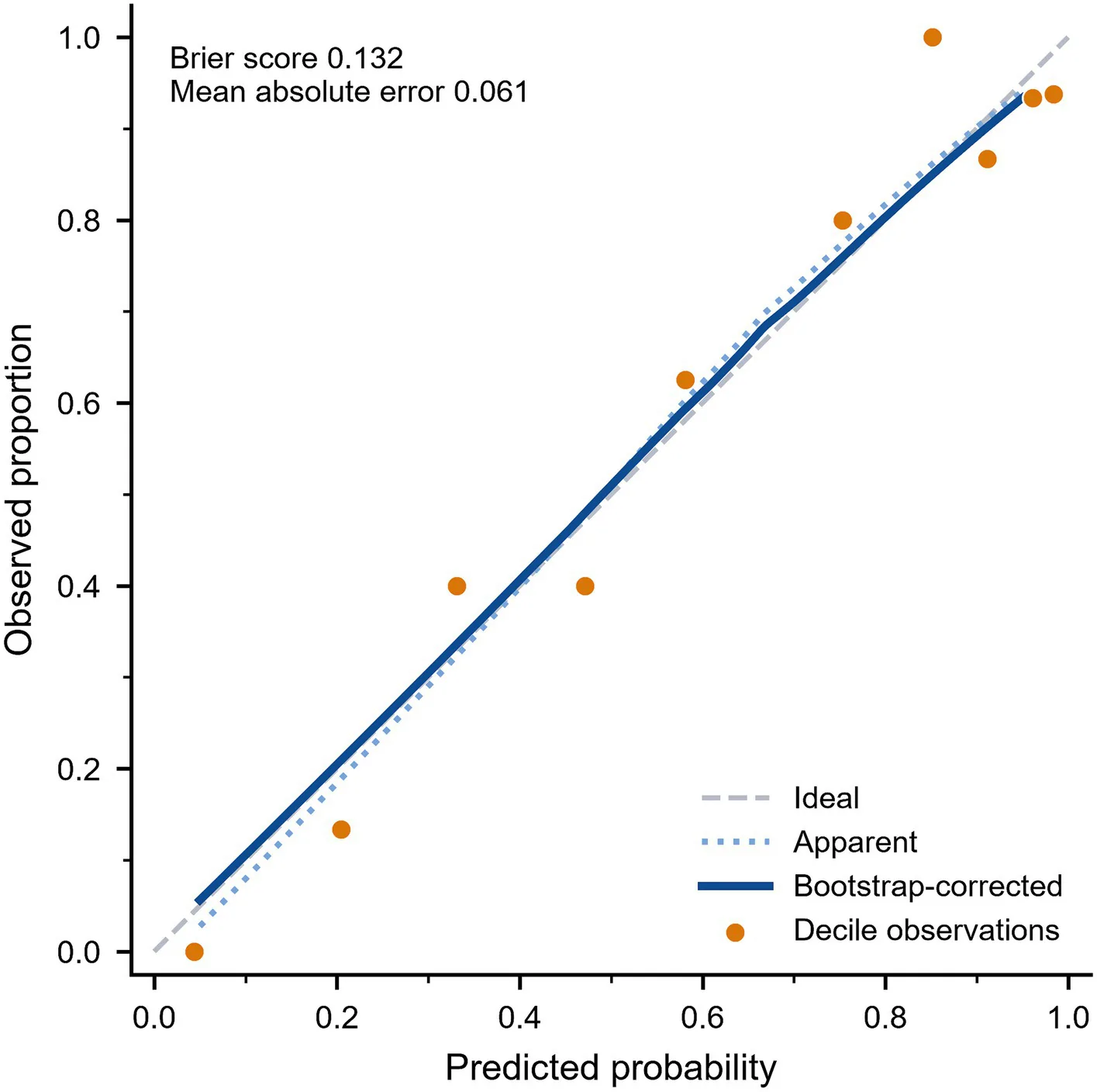
Internal calibration of the PNI-extended model. The orange points represent the observed CI proportions by predicted risk decile. After 1,000 bootstrap resamples, the Brier score was 0.132, and the mean absolute calibration error was 0.061.

## 4 Discussion

Lower PNI remained independently associated with prevalent CI after adjusting for age, sex, and educational attainment. This statistical association must be distinguished from incremental clinical utility. The PNI-extended model had high apparent discrimination, but the majority of discrimination was already present in the base clinical model, and the increase in the AUC after adding PNI was small and non-significant. Exploratory decision-curve analysis and internal bootstrap assessment did not establish clinical usefulness or transportability, and the cross-sectional design did not support prediction of future cognitive decline.

The observed direction is biologically plausible, but the PNI should not be interpreted as a specific measure of nutritional status. Albumin levels are affected by inflammation, hepatic synthesis, hemodilution, renal or gastrointestinal losses, venous congestion, and overall HF severity independently of dietary intake or nutritional reserves (18, 19). Lymphocyte count is similarly influenced by immune activation, infection, medications, and physiologic stress (20). These processes may also be associated with cerebral hypoperfusion, vascular dysfunction, neuroinflammation, and reduced cognitive reserve (6–8, 19–21). The PNI may therefore capture a composite of nutritional, inflammatory, immune, congestion-related, and disease-severity signals rather than nutrition alone.

CI was present in 60.8% of this sample, which is higher than the pooled prevalence of approximately 43% in a recent global meta-analysis (1), but falls within the range observed across contemporary HF cohorts and reviews (2, 4, 7, 8). The difference may reflect an older or more clinically complex study population, the use of the sensitive MoCA threshold, or local referral patterns. The high prevalence also indicates that probability thresholds and predictive values may not apply in lower-prevalence settings. Patients with CI had a higher median LVEF and a numerically greater proportion of HFpEF. This apparently counterintuitive finding may partly reflect the older age and greater proportion of women in the CI group, characteristics frequently associated with HFpEF, in addition to residual differences in comorbidities, HF duration, and treatment. No evidence of effect modification by the HF phenotype was detected; however, the interaction analysis was limited by small subgroup sizes. The cross-sectional LVEF difference should therefore not be interpreted as evidence that higher LVEF contributes to CI.

The albumin sensitivity analysis strengthens this interpretation. Albumin remained independently associated with prevalent CI after adjusting for age, sex, and educational attainment (OR: 0.362 per 5-g/L increase; *p* = 0.007). The PNI did not significantly outperform albumin either as a single marker (AUC: 0.713 vs. 0.693; DeLong *p* = 0.497) or when added separately to the same clinical covariates (AUC: 0.882 vs. 0.887; DeLong *p* = 0.588). Thus, the present data do not show that combining serum albumin with lymphocyte count provides greater discrimination than serum albumin alone. Because serum albumin and lymphocyte count are algebraic components of the PNI, they were evaluated in alternative rather than simultaneous models; the results should not be interpreted as demonstrating independent causal effects.

Clinically, the PNI is attractive because it uses routine laboratory measurements and requires no additional procedure. Nevertheless, the present findings do not justify using PNI as a stand-alone diagnostic or screening test, nor do they demonstrate incremental clinical value beyond age, sex, educational attainment, and albumin. The PNI may be viewed as an exploratory correlate that could prompt formal cognitive assessment in research settings, but the MoCA and comprehensive neuropsychological assessment remain necessary for cognitive evaluation (2, 5, 6, 22). All discrimination, calibration, and decision-curve estimates were derived from an internal assessment of the same cohort; transportability and clinical utility require evaluation in independent prospective cohorts.

This study has several limitations. First, its cross-sectional design precludes conclusions about temporality, causality, or future cognitive decline. Second, the study included a modest sample recruited from a single center, and selection bias arising from recruitment and exclusions cannot be excluded. Third, residual confounding factors such as depression, frailty, prior stroke, sleep disorders, socioeconomic status, medication use, HF duration, and other measures of disease severity remain possible. The PNI was derived from single measurements of serum albumin and lymphocyte count, and no direct nutritional assessments, such as dietary intake, recent weight loss, the Mini Nutritional Assessment, or the Global Leadership Initiative on Malnutrition (GLIM) criteria, were available; consequently, a nutrition-specific interpretation is not supported. Although the HF phenotype was explored, the study had limited power to detect phenotype-specific associations or interactions. Finally, despite bootstrap optimism correction, the model was developed and internally assessed in the same relatively small cohort. Temporal and external validation are required before performance or clinical utility can be generalized.

## 5 Conclusion

Lower PNI was independently associated with prevalent MoCA-defined CI among patients with HF. This association does not establish incremental clinical utility: the increase in AUC after adding PNI to age, sex, and educational attainment was small and non-significant, the decision-curve analysis was exploratory, and the PNI did not outperform albumin in the sensitivity analysis. Prospective multicenter external validation and direct nutritional assessment are required before the PNI can be considered for clinical classification or screening strategies.

## Data Availability

The original contributions presented in the study are included in the article/supplementary material, further inquiries can be directed to the corresponding authors.
